# Preference-based measure of health-related quality of life and its determinants in sickle cell disease in Nigeria

**DOI:** 10.1371/journal.pone.0223043

**Published:** 2019-11-18

**Authors:** Adedokun Oluwafemi Ojelabi, Afolabi Elijah Bamgboye, Jonathan Ling

**Affiliations:** 1 University of Ibadan, Ibadan, Nigeria; 2 School of Nursing and Health Sciences, University of Sunderland, Sunderland, United Kingdom; Gettysburg College, UNITED STATES

## Abstract

**Background:**

Health-related quality of life (HRQL) and economic burden are important issues for people with sickle cell disease (SCD) owing to better survival due to medical advances. Preference-based or utility information is necessary to make informed economic decisions on treatment and alternative therapies. This study aimed to assess preference-based measures of HRQL in sickle cell patients.

**Methods and findings:**

Data were collected from two SCD outpatient clinics in Ibadan, Nigeria. A standard algorithm was used to derive utility scores, and measure SF-6D from the SF-36. A multivariate regression model was used to assess predictors and their impact. A combination of socio-demographic, bio-physiological and psychosocial variables predicted utility score in people with SCD. Socio-demographic and bio-physiological factors explained 7.5% and 17.9% of the variance respectively, while psychosocial factors explained 4.9%. Women had lower utility scores with a small effect size (d = 0.17). Utility score increased with level of education but decreased with age, anxiety, frequency of pain episodes and number of co-morbidities.

**Conclusions:**

Utility score in SCD was low indicating a substantial impact of the disease on HRQL of patients and the value they place on their health state due to the limitations they experienced. Interventions should include both clinical and psychosocial approach to help in improving their quality of life of the patients.

## Introduction

Worldwide, approximately 1,000 children are born daily with sickle cell disease[1–3]. The disease is the most common genetic disease in sub-Saharan Africa accounting for over 5% of under-five mortality in Africa [2,4,5]. The clinical manifestation of SCD range from mild to very severe symptoms across the ages. Genotype, the volume of foetal haemoglobin (HbF) and comorbidities have been suggested as factors responsible for degree of severity[6–10]. Some of the acute and chronic clinical manifestations include painful crisis, vaso-occlusive episodes, stroke, anaemia, hand-foot syndrome, jaundice, frequent infections, delayed growth, vision problems, aplastic crisis, acute chest syndrome, leg ulcers, priapism, pulmonary hypertension and organ damage [9,11–16]. Though survival has increased in the last four decades such that people with SCD can now live into the fifth decade of life [17–20], the impact on the HRQL of the affected individual is substantial[21–23]. Consequently, studies have recognised the importance of HRQL and cost-effectiveness of interventions as outcome measures in addition to survival and morbidity [24].

The common and widely accepted means of measuring HRQL is through psychometric, non-preference-based, methods. Such methods do not provide utilities to assess preferences of the patient or to carry out cost-effectiveness analysis of therapy.While non-preference-based methods have been used to investigate HRQL in SCD[23,25–28], preference-based studies have been lacking. Non-preference-based methods focus on functions in the domains of health assessed, while preference-based or utility measures explore how patients (or the general population) value experiencing a given health state that is defined by functioning and well-being in those domains. The utility approach focuses on the respondents’ valuations of their (health) states whereby outcomes are measured in terms of the preferences that individuals express for being in particular states. The measures combine a descriptive component with the respective valuation component, where the valuation part reflects the values that a society attaches to different health states[29]. While non-preference-based measures aim to measure health (or change in health) perceived by individuals, the objective of a utility measure is to value health states[29].

Quality-of-life measures provide ratings or rankings of health and life[30]. Utility measures move the measurement of quality of life from rankings to judgments of the worth or value of life with a given state of health. The utility approach to healthcare is based on modern utility theory–a model of rational decision-making under uncertainty as posited by von Neuman and Morgenstern [31]. They argued that utilities are indicators of an individual’s preference for particular outcomes or health state under conditions of uncertainty. It has been suggested that the utility approach is a viable alternative for investigators to use in measuring health-related quality of life [32,33].

Utility measures provide single scores across domains of health that range from 0 to 1, (0 =“dead”, and 1 = “perfect health”). A single utility score is important for a variety of reasons one of which is to assess cost effectiveness of interventions with the aim of making informed decisions on the use of healthcare resources [24]. In addition, utility can be used to compare different therapies, such as in comparative effectiveness research[34]. For example, an intervention that produces a difference in utility of 0.03–0.04 is of clinical importance[35] because utilities attach numerical value to the strength of individual, population or society preference for different health outcomes[36]. Furthermore, utility can be used to measure overall health impact to aid decision making. Moreover, utility scores can be combined with life-expectancy estimates to obtain the quality-adjusted life years (QALYs) which is useful for economic evaluations of cost and effectiveness. Health economic analysis of new medical interventions require preference-based weighted measures of quality of life utilities to estimate QALYs[37]. QALYs are valuable in economic evaluations because they incorporate the gained life years as well as the quality of life years to enhance informed decision making. QALYs are the preferred outcome in cost-effectiveness studies which enables comparisons between treatment alternatives [38].

Moreover, utility values are useful in quantifying signs and symptoms related to healthcare, and desirability of a particular disease state according to how a patient perceives his or her life[39,40]. This is useful in evidence-based medicine where best clinical decision-making is a product of high quality clinical research, clinician’s expertise and experience and the patient’s values, desires and perspectives[41]. Furthermore, the efficacy of a medical intervention in improving patient quality of life can be measured by examining improvement in utility values[42].

Utilities are intended for use in any population or disease group [43] and thusare relevant in SCD. However, to our knowledge studies on determinants of utilities in SCD have been lacking in existing literature. Anie et al [36] examined the relationship between self-reported pain, mood and utility in adults with SCD during and after hospital admission. In contrast, our study examined biological, psychosocial and sociodemographic determinants of utilities in adults with SCD who visited hospital for routine medical appointments. The aims of this study therefore were (1) to describe HRQL using a preference-based approach in SCD patients in Nigeria and (2) to investigate socio-demographic, bio-physiological and psychosocial predictors of utility score in the study population. The results can be used to compare the burden of SCD to other disease conditions as well as form an important parameter in cost utility analyses.

## Methods

### Sample and design

Data for this cross-sectional study were collected at the outpatient units of two sickle cell clinics in Ibadan, the University College Hospital (UCH) and Adeoyo General Hospital. We used a convenience sampling method whereby participants were recruited as they arrived at the clinics. The purpose of the research was explained to them and were told of their rights to participate or to withdraw at anytime during the exercise. They were given informed consent forms to read and sign if they agreed to participate. Respondents were made up of 200 adults diagnosed with SCD, aged 18 years and older (see Table 1).

**Table 1:** Demographic information of the participants (n = 200).

Variables	Frequency	Percentage
*Gender*		
Male	83	41.5
Female	117	58.5
*Genotype*		
HbSS	170	85
HbSC	30	15
*Marital Status*		
Never Married	151	75.5
Married	41	20.5
Other (separated, divorced, widowed)	8	4
*Education*		
Primary	19	9.5
Secondary	97	48.5
Post-secondary	84	42
*Employment status*		
Full employment	127	63.5
Part-time employment	30	15
Not employed	43	21.5
*Income level*		
Below minimum wage	167	83.5
≥ Minimum wage	33	15.5
*Living situation*		
Living alone	18	9
Living with others	182	91
*Have a confidant*		
No	29	14.5
Yes	171	85.5
*Religion*		
Christian	105	52.5
Muslim	94	47
Traditional	1	0.5
*Co-morbidity*		
No	135	67.5
Yes	65	32.5
**Co-morbid disease**	**Frequency**	Percent
Asthma	8	4%
Arthritis	11	5.50%
Diabetes	6	3%
Epilepsy	3	1.50%
Heart disease	3	1.50%
High blood pressure	16	8%
Leg ulcers	25	12.50%
Lung disease	9	4.50%
Pneumonia	13	6.50%
Priapism	17	8.50%
Stroke	3	1.50%
Others	5	2.50%

### Ethical approval

The Ethics Board of the University of Ibadan/University College Hospital Ethics Committee and the Research Ethics Committee of the University of Sunderland granted approval for this study.

### Instruments

A personal information questionnaire was administered along with other instruments such as the SF-36, GAD-7 and PHQ-9. The personal information questionnaire was designed to elicit information on socio-demographic variables and self-report of hospitalisation, blood transfusion, pain episodes in the last six months and co-morbidities. Self-reports of co-morbidities tend to be accurate, and therefore represent a reliable and valid measure of actual co-morbidity [44–47]. Eleven co-morbidities related to SCD identified in literature[48–52] were specified in this instrument. They were asthma, arthritis, diabetes, epilepsy, heart disease, high blood pressure, leg ulcers, lung disease, pneumonia, priapism and stroke. Participants were asked to indicate which of these they had or had been informed by their doctors that they had. Participants were also asked to mention any other diseases they had that were not listed. The co-morbidities were scored for individual respondents (1–12) depending on number of comorbidities. This was re-coded as 0, 1–2, and 3 or more to reduce skewness in the distribution.

Assessment of Utility: Utility could be derived through the direct method which consists of eliciting information from individuals through rating scale (RS), standard gamble (SG) or time trade-off (TTO) techniques. The indirect approach uses multi-attribute utility scales. This involves the use of a questionnaire. Individuals complete the questionnaire to describe their health state, these descriptions are converted to index scores using societal valuations. Health state valuations are normally derived from a representative sample of the general population[53]

This study employed the indirect method whereby individuals completed questionnaires to describe health scale which are then converted to index scores using social valuations [43]. The Short Form (SF-36) version 1 health survey [54]is a widely used general HRQL measure which has been validated across a wide variety of age, race, disease populations including SCD with acceptable psychometric properties [25,55–59]. The SF-36 contains 36 questions aggregated into 8 domains namely physical function, role limitation, social function, bodily pain, mental health and vitality, role physical and general health. These domains are summarised into two health components, the Physical Component scores (PCS) and the Mental Component score (MCS). The utility measure, SF-6D was derived from 11 of the 36 questions of the SF-36 instrument which include six dimensions (physical function, role limitation, social function, bodily pain, mental health and vitality) that defined 18,000 health states [60]. The scoring algorithm to derive SF-6D from SF-36[61–63]was requested and obtained from Sheffield University (http://www.sheffield.ac.uk/scharr/sections/heds/mvh/noncommercial).The algorithm used preference weights obtained from a sample of the UK general population to generate the SF-6D utility score in the UK population. Utility scores ranged from 0.3 to 1.00 where 1.00 represents “full health”.[64]. The psychometric properties of SF-6D have been investigated and reported to be acceptable[43].

Assessment of anxiety: We used the generalised anxiety disorder (GAD-7) instrument to measure anxiety in the population. GAD-7[65]is a 7-item scale developed for screening generalised anxiety disorder in line with the *Diagnostic and Statistical Manual of Mental Disorders* Fourth Edition (DSM-IV)[66] diagnoses. Respondents were asked to rate their experience in the last two weeks with respect to how they were bothered by symptoms of anxiety. Rating options were ‘not at all’, ‘several days’, ‘more than half the days’, ‘nearly every day’. The ratings were allocated scores of 0,1,2,3 respectively. The GAD-7 total score ranges from 0 to 21. Cut points of 5, 10, and 15 were interpreted as representing mild, moderate and severe levels of anxiety. The instrument has good psychometric properties [65] and has been used to measure anxiety in SCD populations in Brazil [67] and the USA [68].

Assessment of depression: The Patient Health Questionnaire (PHQ) was used to assess depression in the population. The PHQ-9[69] is a subscale of the PHQ designed to assess depression in patients. The instrument has been established to be reliable and valid instrument for screening depressive disorder according to the DSM-IV diagnoses [69]. Patients were asked to rate how often they had been bothered by each of the depressive symptoms in the last two weeks. Experiences were rated from 0, ‘not at all’ to 3, ‘nearly every day’. The total possible score ranges from 0 to 27. Symptoms of depression were assumed to be minimal for score < 5, mild = 5–9, moderate = 10–14, and severe, ≥15 [70]. PHQ-9 has been shown to be equal or superior to other measures of depression [71–73]. The psychometric properties of PHQ-9 have been well documented [70] and have been used in measuring depression in SCD [67,68,74,75]. Items left blank (missing data) were filled with the mean scores of the completed items provided missing items were less than 20%, otherwise the item was treated as completely missing for the individual; this approach was considered to have a lower risk of missing persons with depression, anxiety or somatisation [70]. However, no item or respondent in this study had up to 20% missing values.

Assessment of pain and hospital admissions: The measures were made up of events in the life of the participants in the last six months prior to data collection. Participants were asked to indicate the number of times they have been admitted into the hospital in the last 6 months on a scale of 0 (none) to 4 (more than three times). In the same vein, they were asked to state how many times they had had pain episodes in the last 6 months on the same scale.

Reliability: Though the psychometric properties of these instruments have been established in different studies, populations and countries, However, researchers have suggested that investigators should not rely on published reliability estimates because alpha is a property obtained from a specific sample of the population being tested [76,77]. Reliability was investigated using the Cronbach alpha [78].

### Statistical analysis

Statistical analyses were performed using SPSS version 24 (SPSS, Inc., Chicago). Descriptive statistics were computed for all the variables. Means and standard deviations were obtained for continuous variables, and frequencies and percentages for the categorical variables. Utility scores were presented as mean values, standard deviations (SD) and 95% confidence interval (CI). The Shapiro-Wilk test indicated that the utility score distribution in the population was normal (Shapiro-Wilk = 0.994, p = 0.601). We also report utility score stratified by socio-demographic variables. Student’s t-test was used to compare utility between two independent variables and one-way analysis of variance (ANOVA) with post-hoc analysis was used in variables with more than two categories. We examined the collinearity statistics to identify possible presence of multi-collinearity. The Tolerance statistic (range 0.36–0.93) and the variance inflation factor (VIF, range 1.1–4.5) statistic showed that multi-collinearity was not present among the independent variables. Possible associations between utility score and other variables was investigated using bivariate correlation. A hierarchical regression was used to test predictors. The independent variables were selected based on previous studies [27,48,79–81]. The variables were entered in three blocks based on order employed in similar studies [82,83]. The first block was made up of socio-demographic variables namely age, gender, marital status, education, employment and income. The second block consisted of biological variables such as genotype, number of co-morbidities, frequency of pain episode and hospital admission. The third block included psychosocial variables, namely anxiety and depression. Statistical significance was set at 5%. To measure substantive significance, we calculated and classified effect sizes as small (0.3), medium (0.5) or large (0.8) according to Cohen’s recommendation [84]. The 95% confidence interval (CI) was also computed for variables of interest.

## Results

The mean age of participants was 27.9 years (SD: 6.95) with 58.5% of them female (see Table 1). Single participants or Never married constituted 75.5% of the sample, while 20.5% were married, with 4% either divorced or widowed. Eighty-five percent of the participants had the HbSS genotype while 15% were HbSC. There was no Hb thalassemia in the population. The educational profile showed only 9.5% had below secondary education and 42.2% have tertiary education. Only 36% indicated that they had full time or part time employment and about 93% were either living with relatives or friends. Most of the participants reported that they have confidants (85.5%) and 19% percent have children. About one-third (32.5%) of the participants indicated that they had been diagnosed with co-morbidities. The most prevalent co-morbidity was leg ulcers (12.5%) followed by priapism (8.5%) and high blood pressure (8%). Other identified co-morbidities were mostly pain-related, rheumatism, chest pain and back pain.

### Sample characteristics

The means, standard deviations and confidence intervals of variables for SF-6D, GAD-7 and PHQ-9 were calculated (Table 2). The mean utility score in the population was 0.65 (SD: 0.12; range: 0.310–0.965). The reliabilities of GAD-7 and PHQ-9 were acceptable, (Cronbach- α, 0.90 and 0.82, respectively). The mean anxiety in the population was minimal (GAD-7 < 5) while depression was mild (5 <PHQ-9 < 10). A frequency analysis showed that 12% had moderate to severe anxiety while 19% had moderate to severe depression.

**Table 2:** Descriptive analysis.

*Instruments*	Variable	Mean	SD	95% CI	Reliability
SF-6D (Derived from SF-36)	Utility	0.65	0.12	0.63–0.67	
GAD-7	Anxiety	3.86	3.91	3.32–4.40	0.902
PHQ-9	Depression	5.14	4.70	4.49–5.79	0.824
Questionnaire	Pain frequency	2.37	1.19	2.20–2.53	
	Hospital admission	1.495	1.147	1.34–1.66	

The utility score was lower for those who had co-morbidities than those who did not (see Table 3); this difference was statistically significant (p = 0.001) with an effect size of 0.63. Female gender also had lower utility score but not statistically significant (p = 0.179). There were more patients with HbSS than HbSC genotypes but the difference in their utility scores was not statistically significant (p = 0.894). Those living alone reported lower utility than those living with others such as relatives or friends and thus probably enjoyed support, however this difference was not statistically significant (p = 0.227).

**Table 3:** Group differences and effects sizes.

**Groups**	**N**	**Mean**	**SD**	**t-value**	**df**	**95% CI**	**p-value**	**Cohen’s (d)**
**Gender**								
Male	83	0.66	0.11	1.35	198	0.64–0.69	0.179	0.17
Female	117	0.64	0.12			0.62–0.66		
**Living situation**								
Alone	18	0.62	0.08	1.213	198	0.58–0.65	0.227	0.3
With others	182	0.65	0.12			0.64–0.67		
**Confidants**								
Yes	171	0.651	0.11	0.137	198	0.60–0.70	0.891	0.02
No	29	0.647	0.13			0.63–0.67		
**Genotype**								
SS	170	0.651	0.12	0.133	198	0.63–0.67	0.894	0.03
SC	30	0.648	0.12			0.61–0.69		
**Co-morbidity**								
No	135	0.67	0.12	4.338**	198	0.65–0.69	<0.001	0.63
Yes	65	0.6	0.1			0.58–0.63		
**Groups**	**N**	**Mean**	**SD**	**F-value**	**df**	**95% CI**	**p-value**	**Cohen’s (d)**
**Marital Status**				1.141	197		0.322	0.12
Never married	151	0.66	0.12			0.64–0.68		
Married	41	0.63	0.09			0.61–0.66		
Others (divorced, separated, widowed)	8	0.61	0.12			0.53–0.69		
**Education**				2.415	197		0.092	0.16
≤ Primary	19	0.6	0.12			0.55–0.65		
Secondary	97	0.65	0.11			0.63–0.67		
Post-secondary	84	0.66	0.12			0.64–0.67		
**Employment**				0.313	197		0.732	0.06
Full-Time	43	0.66	0.11			0.63–0.67		
Part-time	30	0.64	0.1			0.60–0.68		
Not employed	127	0.65	0.12			0.63–0.70		

** p< 0.01

### Bivariate relationships

The correlation analysis revealed that utility values decreased with an increase in number of co-morbidities (r = -0.32, p<0.001), depression (-0.34, p<0.001), anxiety (r = -0.42, p<0.001, pain frequency (r = -0.31, p<0.001) and hospital admission (r = -0.16, p<0.026) but exhibited a positive relationship with level of education (r = 0.14, p = 0.049). The association with age was positive but not statistically significant (r = 0.14, p = 0.054).

### Multivariate analysis

All the independent variables explained 30.3% of the variation (see Table 4). The socio-demographic factors alone accounted for 7.5%, the predictors were age, education and employment. This increased to 25.4% when the bio-physiological variables were added. Frequency of pain, number of co-morbidities, age and level of education were associated with utility score. Psychosocial variables accounted for an additional 4.9% where anxiety was negatively associated with health utility. Increase in anxiety, frequency of pain and number of co-morbidities led to a reduced utility value while higher level of education positively predicted utility score.

**Table 4:** Predictors of health utility score.

Variables	BUnstandardized	SE (B)	B Standardized	Statistic (t)	p-value	R Sqrd	F-change	Sig. F-change
Block 1				2.598***	0.019	0.075	2.598	0.019
Constant	0.596	0.057		10.515	0.000			
Age	-0.033	0.015	-0.169*	-2.416	0.033			
Gender	-0.021	0.017	-0.091	-1.295	0.197			
Marital-status	0.003	0.017	0.015	-0.186	0.853			
Education	0.035	0.013	0.193**	2.632	0.009			
Employment	0.042	0.02	0.294*	2.131	0.034			
Income	-0.036	0.022	-0.237	-1.671	0.096			
Block 2				6.45	0	0.254	11.389	< 0.001
Constant	0.655	0.058		11.383	0			
Age	-0.031	0.015	-0.159*	-2.139	0.034			
Gender	-0.01	0.015	-0.044	-0.683	0.496			
Marital-status	-0.003	0.015	-0.014	-0.179	0.858			
Education	0.048	0.012	0.263**	3.895	0			
Employment	0.022	0.018	0.158	1.242	0.216			
Income	-0.012	0.02	-0.079	-0.606	0.545			
Genotype	0.009	0.021	0.029	0.441	0.66			
Hospital_admission	-0.006	0.007	-0.059	-0.873	0.384			
Pain-frequency	-0.027	0.007	-0.280**	-4.186	0			
Num-co-morbidity	-0.05	0.013	-0.272**	-3.853	0			
Block 3				6.76	0	0.303	9.407	<0.001
Constant	0.707	0.058		12.212	0			
Age	-0.023	0.014	-0.118	-1.611	0.109			
Gender	-0.006	0.015	-0.025	-0.397	0.692			
Marital-status	-0.008	0.015	-0.039	-0.52	0.603			
Education	0.044	0.012	0.241**	3.645	0			
Employment	0.027	0.018	0.189	1.509	0.133			
Income	-0.017	0.02	-0.11	-0.849	0.397			
Genotype	0.002	0.021	0.007	0.108	0.914			
Hospital_admmission	-0.002	0.007	-0.016	-0.24	0.811			
Pain-frequency	-0.028	0.006	-0.284**	-4.368	0			
Num-co-morbidity Anxiety	-0.029	0.014	-0.156*	-1.997	0.047			
Depression	-0.037	0.014	-0.229*	-2.604	0.01			
	-0.005	0.011	-0.043	-0.481	0.631			

*P<0.05

** P<0.01

*** = F statistic

## Discussion

Health utilities are designed to investigate HRQL across domains of health to provide a single score that incorporates how patients’ (or the general population) value experiencing a given health state that is defined by level of functioning and well-being in those domains. While the source of the values used to initially develop these measures was the general population, such scores are important to measure the burden of diseases. The mean utility score for SCD patients, in our study, (0.65±0.12) compares with the utility score (0.66± 0.26) reported for people with haemochromatosis [85] and (0.66±0.14) reported for people with age-related macular degeneration[86] but much lower than the utility score (0.75±0.14) reported in adults with type 1 diabetes [87], and that for overweight and obese women with urinary incontinence (0.75±0.10) [88], and those with chronic kidney disease (0.67±0.13)[89].Anie *et al*, [36] reported a utility score of 0.39±0.40 for SCD patients on admission for pain which improved to 0.65±0.29 which is similar to our result. Lower utility values indicate that SCD can substantially diminish the HRQL of the affected individual. However, these comparisons should be interpreted with caution as the studies used different methods to derive their respective utility score. For example, the study on haemochromatosis[85] used the Assessment of Quality of life 4D(AQOLD-4D), the study by Anie *et al*[36] used the EuroQol EQ-5D.

The lower utility scores in women than men found in this study might not be statistically significant, more information is needed to establish if this difference is of clinical importance. Similar conclusion may be applicable to the difference between those living alone and those living with others as it has been suggested that a difference of 0.03 could be clinically important [90,91]. The finding of a significant difference between those who had at least one co-morbidity compared to those who had none is worthy of comment. SCD patients are a vulnerable, chronically ill population with a high prevalence of co-morbidities which contributed to reduced quality of life in chronic diseases[92].

Age was negatively associated with utility, as expected, because SCD patients are exposed to increased clinical complications as they grow older.This supports findings from a general UK population where utility score was reported to decrease as age increased[93]due to age-dependent end-organ dysfunction associated with the disease. Before we entered the psychosocial variables into the regression model, age was a significant predictor of utility score, however, the statistical significance disappeared when anxiety and depression were added into the model. We do not know whether age is a confounder in this study especially because a positive relationship was found between age and anxiety. The negative associations of anxiety and depression with health utility found in this study, support previous studies where these variables were reported to associate negatively with HRQL in SCD[67,81,94,95]. The multivariate regression modelling showed that pain frequency, anxiety, number of co-morbidities and education were strong predictors of health utility score. In terms of the direction of effects, these impacts were as expected. For example, anxiety included items that measure mental health while pain has been presented as the hallmark of SCD[36,96,97] and is responsible for emergency visits [96]. Therefore, their increase or severity was found to associate with decreased utility score. This is a pointer to the need to approach clinical management of SCD taking a multidisciplinary perspective so as to ensure better quality of life for the patients. For example, in addition to interventions that focus on biological and physiological variables, routine investigation of patients’ psychiatric status should be integrated into the disease management protocol.

### Clinical implications

Table 4 shows that the beta coefficients ranged between -0.01 and 0.04 on the 0–1 health utility score. According to Walters and Brazier[90], the minimally important difference ranges between 0.01 and 0.05 with a weighted average of 0.03. Khanna[91] also reported a minimally important difference of 0.03 in systemic sclerosis. All the predictors had the unstandardized beta coefficients with absolute values equal or greater than 0.03 indicating that beyond statistical significance, they may be of clinical importance in the management of SCD as well as to enhance the utility score measure of health-related quality of life. However, more information is needed to establish this assertion.

### Limitations

Owing to the cross-sectional nature of the design it was not possible to definitively establish a causal relationship. Also, the study relied on self-reported co-morbid conditions, although previous studies have established high agreement between self-reported co-morbidities and medical records abstractions [44–47,98]. The authors did not examine medical records to validate patients’ reports of their hospital admissions and pain episodes which could be understated or overstated due to recall errors. SF-6D has been reported to have a floor effect and different derivations of utility measures have been reported to yield different results [87,99]. However, SF-6D has been reported to be relatively better in discriminating between health states [87,100]. Our analysis was based on preference weights derived from the UK population because such weighs do not exist in the local population, We are therefore cautious in our interpretations because the standard of living, access to medical facilities and life expectancy are better in the UK. This work could be replicated using different preference-based measures. Further research could also include a longitudinal study of utility in people with SCD using both direct and derived methods. There is also the need to compare direct and derived methods in SCD as well as a comparison of the performance of the different derivation like SF-6D, EQ-5D, HUI3 and so on.

### Conclusion

Health utilities are important values necessary to understand the preferences of an individual for different health outcomes and when combined with survival estimates can be employed in cost utility analysis of medical treatment. Study of health utilities has been lacking in SCD,therefore our findings provide an important contribution to knowledge. This study has shown that utility decreases with age, increasing co-morbidities, painful episodes and anxiety;a multidisciplinary approach is therefore required in the management of the disease. In addition, clinical interventions to ameliorate the painful episodic exacerbations of SCD patients and to manage the disease should also consider the influence of co-morbid conditions. The present study suggests that level of education predicted better utility, although there is need for further studies to establish this relationship as there could be other factors influencing this relationship. It is however recommended that SCD patients, especially in sub-Saharan Africa should be encouraged to enrol in open and distance learning programmes which gives them the opportunity to study at their own pace. As SCD affects schooling, educational policy could be designed to give better opportunities to people with SCD; we believe this could potentially enhance their quality of life.

## Supporting information

S1 Table Sf6d from sf36. (XLSX)

S2 Table Support dataset. (XLSX)

## References

[1] Aliyu ZY, Gordeuk V, Sachdev V, Babadoko A, Mamman AI, Akpanpe P, et al Prevalence and risk factors for pulmonary artery systolic hypertension among sickle cell disease patients in Nigeria. Am J Hematol. 2008; 485–490. 10.1002/ajh.21162 18306362 PMC3415268

[2] W H O. Report by the Secretariat of the Fifty-Ninth World Health Assembly A59/9. Rep Secr Fifty-ninth World Heal Assem A59/9. 2006.

[3] Weatherall DJ, Clegg JB. Inherited haemoglobin disorders: an increasing global health problem. Bull World Health Organ. 2001;79: 704–712. 11545326 PMC2566499

[4] Modell B, Darlison M. Global epidemiology of haemoglobin disorders and derived service indicators. Bull World Health Organ. 2008;86: 480–487. 10.2471/BLT.06.036673 18568278 PMC2647473

[5] United Nations. General Assembly Resolution on Recognition of Sickle Cell Anaemia as a Public Health Problem. A/RES/63/237. 2008;1: 2005–2006. 10.1093/oxfordhb/9780199560103.003.0005

[6] Ballas SK, Lewis CN, Noone AM, Krasnow SH, Kamarulzaman E, Burka ER. Clinical, hematological, and biochemical features of Hb SC disease. Am J Hematol. 1982;13: 37–51. 10.1002/ajh.2830130106 7137165

[7] Powars D, Hiti A. Sickle cell anemia: βS gene cluster haplotypes as genetic markers for severe disease expression. Am J Dis Child. 1993;147: 1197–1202. 10.1001/archpedi.1993.02160350071011 8237915

[8] Thomas PW, Higgs DR, Serjeant GR. Benign clinical course in homozygous sickle cell disease: a search for predictors. J Clin Epidemiol. 1997;50: 121–126. 10.1016/s0895-4356(96)00320-4 9120504

[9] Ohene-Frempong K, Weiner SJ, Sleeper LA, Miller ST, Embury S, Moohr JW, et al Cerebrovascular accidents in sickle cell disease: rates and risk factors. Blood. 1998;91: 288–294. 9414296

[10] Ashley-Koch A., Yang Q. and Olney RS. Sickle Hemoglobin (Hb S) Allele and Sickle Cell Disease: A HuGE Review. Am J Epidemiol Huge Genome Epidemiol Rev. 2000;151: 839–845. 10.1093/oxfordjournals.aje.a010288 10791557

[11] Castro O, Brambilla DJ, Thorington B, Reindorf CA, Scott RB, Gillette P, et al The acute chest syndrome in sickle cell disease: incidence and risk factors. The Cooperative Study of Sickle Cell Disease. Blood. 1994;84: 643–649. 7517723

[12] Oliveira CC de, Ciasca SM, Moura-Ribeiro M. Stroke in patients with sickle cell disease: clinical and neurological aspects. Arq Neuropsiquiatr. 2008;66: 30–33. 10.1590/s0004-282x2008000100008 18392410

[13] Powars DR. Sickle cell anemia and major organ failure. Hemoglobin. 1990;14: 573–598. 10.3109/03630269009046967 2101835

[14] Scheinman JI. Sickle cell disease and the kidney. Nat Rev Nephrol. 2009;5: 78. 10.1038/ncpneph1008 19048000

[15] Ladizinski B, Bazakas A, Mistry N, Alavi A, Sibbald RG, Salcido R. Sickle cell disease and leg ulcers. Adv Skin Wound Care. 2012;25: 420–428. 10.1097/01.ASW.0000419408.37323.0c 22914039

[16] Hernigou P, Galacteros F, Bachir D, Goutallier D. Deformities of the hip in adults who have sickle-cell disease and had avascular necrosis in childhood. A natural history of fifty-two patients. J Bone Joint Surg Am. 1991;73: 81–92. 1985998

[17] Grosse SD, Odame I, Atrash HK, Amendah DD, Piel FB, Williams TN. Sickle cell disease in Africa: a neglected cause of early childhood mortality. Am J Prev Med. 2011;41: S398–S405. 10.1016/j.amepre.2011.09.013 22099364 PMC3708126

[18] Lanzkron S, Carroll CP, Haywood C Jr. Mortality rates and age at death from sickle cell disease: US, 1979–2005. Public Health Rep. 2013;128: 110–116. 10.1177/003335491312800206 23450875 PMC3560868

[19] Platt OS, Brambilla DJ, Rosse WF, Milner PF, Castro O, Steinberg MH, et al Mortality in sickle cell disease—life expectancy and risk factors for early death. N Engl J Med. 1994;330: 1639–1644. 10.1056/NEJM199406093302303 7993409

[20] Ware RE. Is sickle cell anemia a neglected tropical disease? PLoS Negl Trop Dis. 2013;7: e2120 10.1371/journal.pntd.0002120 23750287 PMC3671937

[21] Anie KA, Steptoe A, Bevan DH. Sickle cell disease: Pain, coping and quality of life in a study of adults in the UK. Br J Health Psychol. 2002;7: 331–344. 10.1348/135910702760213715 12614504

[22] Menezes AS de O da P, Len CA, Hilário MOE, Terreri MTRA, Braga JAP. Qualidade de vida em portadores de doença falciforme. Rev Paul Pediatr. 2013;31: 24–29. 10.1590/s0103-05822013000100005 23703040

[23] Caird H, Camic PM, Thomas V. The lives of adults over 30 living with sickle cell disorder. Br J Health Psychol. 2011;16: 542–558. 10.1348/135910710X529278 21722275

[24] van Litsenburg Raphaële R L, Huisman J, Raat H, Kaspers GJL, Gemke RJBJ. Health-related quality of life and utility scores in short-term survivors of pediatric acute lymphoblastic leukemia. Qual Life Res. 2013;22: 677–681. 10.1007/s11136-012-0183-x 22547048 PMC3607731

[25] Asnani M, Lipps G, Reid M. Component structure of the SF-36 in Jamaicans with sickle cell disease. West Indian Med J. 2007;56: 491–497. 18646491

[26] Sogutlu A, Levenson JL, McClish DK, Rosef SD, Smith WR. Somatic symptom burden in adults with sickle cell disease predicts pain, depression, anxiety, health care utilization, and quality of life: the PiSCES project. Psychosomatics. 2011;52: 272–279. 10.1016/j.psym.2011.01.010 21565599

[27] McClish DK, Penberthy LT, Bovbjerg VE, Roberts JD, Aisiku IP, Levenson JL, et al 2005. {Health} related quality of life in sickle cell patients: the {PiSCES} project. Health Qual Life Outcomes. 2005;3: 50 10.1186/1477-7525-3-50 16129027 PMC1253526

[28] Gibson RC, Morgan KAD, Abel WD, Sewell CA, Martin JS, Lowe GA, et al Locus of control, depression and quality of life among persons with sickle cell disease in Jamaica. Psychol Health Med. 2013;18: 451–460. 10.1080/13548506.2012.749353 23324018

[29] Brazier J, Deverill M. A checklist for judging preference‐based measures of health related quality of life: learning from psychometrics. Health Econ. 1999;8: 41–51. 10.1002/(sici)1099-1050(199902)8:1<41::aid-hec395>3.0.co;2-# 10082142

[30] Schuessler KF, Fisher GA. Quality of life research and sociology. Annu Rev Sociol. 1985;11: 129–149.

[31] Von Neumann J, Morgenstern O. Theory of games and economic behavior, 2nd rev. 1947.

[32] Torrance GW. Measurement of health state utilities for economic appraisal: a review. J Health Econ. 1986;5: 1–30. 10.1016/0167-6296(86)90020-2 10311607

[33] Torrance GW. Utility approach to measuring health-related quality of life. J Chronic Dis. 1987;40: 593–603. 10.1016/0021-9681(87)90019-1 3298297

[34] Hays RD, Reeve BB, Smith AW, Clauser SB. Associations of cancer and other chronic medical conditions with SF-6D preference-based scores in Medicare beneficiaries. Qual Life Res. 2014;23: 385–391. 10.1007/s11136-013-0503-9 23990395 PMC3938981

[35] Drummond M. Introducing economic and quality of life measurements into clinical studies. Ann Med. 2001;33: 344–349. 10.3109/07853890109002088 11491193

[36] Anie KA, Grocott H, White L, Dzingina M, Rogers G, Cho G. Patient self-assessment of hospital pain, mood and health-related quality of life in adults with sickle cell disease. BMJ Open. 2012;2: e001274–e001274. 10.1136/bmjopen-2012-001274 22761289 PMC3391376

[37] Gold MR. Cost-effectiveness in health and medicine Oxford university press; 1996.

[38] Wyld M, Morton RL, Hayen A, Howard K, Webster AC. A systematic review and meta-analysis of utility-based quality of life in chronic kidney disease treatments. PLoS Med. 2012;9: e1001307 10.1371/journal.pmed.1001307 22984353 PMC3439392

[39] Brown GC, Sharma S, Brown MM, Kistler J. Utility values and age-related macular degeneration. Arch Ophthalmol. 2000;118: 47–51. 10.1001/archopht.118.1.47 10636413

[40] Sharma S, Brown GC, Brown MM, Hollands H, Robins R, Shah GK. Validity of the time trade-off and standard gamble methods of utility assessment in retinal patients. Br J Ophthalmol. 2002;86: 493–496. 10.1136/bjo.86.5.493 11973240 PMC1771136

[41] Bramlett RE, Bothe AK, Franic DM. Using preference-based measures to assess quality of life in stuttering. J Speech, Lang Hear Res. 2006;49: 381–394. 16671851 10.1044/1092-4388(2006/030)

[42] Brown GC, Brown MM, Sharma S, Beauchamp G, Hollands H. The reproducibility of ophthalmic utility values. Trans Am Ophthalmol Soc. 2001;99: 199 11797307 PMC1359010

[43] Engel L, Bryan S, Evers SMAA, Dirksen CD, Noonan VK, Whitehurst DGT. Exploring psychometric properties of the SF-6D, a preference-based health-related quality of life measure, in the context of spinal cord injury. Qual Life Res. 2014;23: 2383–2393. 10.1007/s11136-014-0677-9 24700379

[44] Bayliss EA, Ellis JL, Steiner JF. Subjective assessments of comorbidity correlate with quality of life health outcomes: initial validation of a comorbidity assessment instrument. Health Qual Life Outcomes. 2005;3: 51 10.1186/1477-7525-3-51 16137329 PMC1208932

[45] Kriegsman DMW, Penninx BWJH, Van Eijk JTM, Boeke AJP, Deeg DJH. Self-reports and general practitioner information on the presence of chronic diseases in community dwelling elderly: a study on the accuracy of patients’ self-reports and on determinants of inaccuracy. J Clin Epidemiol. 1996;49: 1407–1417. 10.1016/s0895-4356(96)00274-0 8970491

[46] Penninx BWJH, Beekman ATF, Ormel J, Kriegsman DMW, Boeke AJP, Van Eijk JTM, et al Psychological status among elderly people with chronic diseases: does type of disease play a part? J Psychosom Res. 1996;40: 521–534. 10.1016/0022-3999(95)00620-6 8803861

[47] Van Den Bos GAM. The burden of chronic diseases in terms of disability, use of health care and healthy life expectancies. Eur J Public Health. 1995;5: 29–34.

[48] Ojelabi A, Graham Y, Ling J. Health-related Quality of Life Predictors in Children and Adolescents with Sickle Cell Disease: A Systematic Review. Int J Trop Dis Heal. 2017;22: 1–14. 10.9734/IJTDH/2017/31954

[49] Konotey-Ahulu FID. The sickle cell diseases: Clinical manifestations including the sickle crisis. Arch Intern Med. 1974;133: 611–619. 4818434

[50] Wrotniak BH, Schall JI, Brault ME, Balmer DF, Stallings VA. Health-related quality of life in children with sickle cell disease using the child health questionnaire. J Pediatr Heal Care. 2014;28: 14–22. 10.1016/j.pedhc.2012.09.004 23140759 PMC4479286

[51] Coleman B, Ellis-Caird H, McGowan J, Benjamin MJ. How sickle cell disease patients experience, understand and explain their pain: An Interpretative Phenomenological Analysis study. Br J Health Psychol. 2016;21: 190–203. 10.1111/bjhp.12157 26333530

[52] Kohne E. Hemoglobinopathies: clinical manifestations, diagnosis, and treatment. Dtsch Ärzteblatt Int. 2011;108: 532–40. 10.3238/arztebl.2011.0532 21886666 PMC3163784

[53] Brazier J, Usherwood T, Harper R, Thomas K. Deriving a preference-based single index from the UK SF-36 Health Survey. J Clin Epidemiol. 1998;51: 1115–1128. 10.1016/s0895-4356(98)00103-6 9817129

[54] Ware Jr JE, Sherbourne CD. The MOS 36-item short-form health survey (SF-36): I. Conceptual framework and item selection. Med Care. 1992; 473–483. 1593914

[55] Shiu ATY, Choi KC, Lee DTF, Yu DSF, Man Ng W. Application of a health-related quality of life conceptual model in community-dwelling older Chinese people with diabetes to understand the relationships among clinical and psychological outcomes. J Diabetes Investig. 2014;5: 677–686. 10.1111/jdi.12198 25422768 PMC4234231

[56] Bullinger M. German translation and psychometric testing of the SF-36 health survey: preliminary results from the IQOLA project. Soc Sci Med. 1995;41: 1359–1366. 10.1016/0277-9536(95)00115-n 8560303

[57] Phaladze NA, Human S, Dlamini SB, Hulela EB, Hadebe IM, Sukati NA, et al Quality of Life and the Concept of “Living Well” With HIV / AIDS in Sub-Saharan Africa. J Nurs Schorlarsh. 2005;37: 120–126. 10.1111/j.1547-5069.2005.00023.x 15960055

[58] Dampier C, LeBeau P, Rhee S, Lieff S, Kesler K, Ballas S, et al Health-related quality of life in adults with sickle cell disease (SCD): A report from the comprehensive sickle cell centers clinical trial consortium. Am J Hematol. 2011;86: 203–205. 10.1002/ajh.21905 21264908 PMC3554393

[59] Asnani MR, Lipps GE, Reid ME. Validation of the SF-36 in Jamaicans with sickle-cell disease. Psychol Health Med. 2009;14: 606–618. 10.1080/13548500903016567 19844839

[60] Brazier J, Roberts J, Deverill M. The estimation of a preference-based measure of health from the SF-36. J Health Econ. 2002;21: 271–292. 10.1016/s0167-6296(01)00130-8 11939242

[61] Brazier JE, Roberts J. The estimation of a preference-based measure of health from the SF-12. Med Care. 2004;42: 851–859. 10.1097/01.mlr.0000135827.18610.0d 15319610

[62] Kharroubi SA, Brazier JE, Roberts J, O’Hagan A. Modelling SF-6D health state preference data using a nonparametric Bayesian method. J Health Econ. 2007;26: 597–612. 10.1016/j.jhealeco.2006.09.002 17069909

[63] McCabe C, Brazier J, Gilks P, Tsuchiya A, Roberts J, O’Hagan A, et al Using rank data to estimate health state utility models. J Health Econ. 2006;25: 418–431. 10.1016/j.jhealeco.2005.07.008 16499981

[64] Walters SJ, Brazier JE. Comparison of the minimally important difference for two health state utility measures: EQ-5D and SF-6D. Qual life Res. 2005;14: 1523–1532. 10.1007/s11136-004-7713-0 16110932

[65] Spitzer RL, Kroenke K, Williams JBW, Löwe B. A brief measure for assessing generalized anxiety disorder: the {GAD}-7. Arch Intern Med. 2006;166: 1092–1097. 10.1001/archinte.166.10.1092 16717171

[66] Edition F. Diagnostic and statistical manual of mental disorders. Am Psychiatric Assoc; 2013.

[67] Mastandréa ÉB, Lucchesi F, Kitayama MMG, Figueiredo MS, Citero V de A. The relationship between genotype, psychiatric symptoms and quality of life in adult patients with sickle cell disease in São Paulo, Brazil: a cross-sectional study. Sao Paulo Med J. 2015;133: 00 10.1590/1516-3180.2015.00171105 26648431 PMC10871801

[68] Treadwell MJ, Barreda F, Kaur K, Gildengorin G. Emotional distress, barriers to care, and health-related quality of life in sickle cell disease. J Clin Outcomes Manag. 2015;22: 10–20.

[69] Kroenke K, Spitzer RL, Williams JBW. The PHQ-9: Validity of a brief depression severity measure. J Gen Intern Med. 2001;16: 606–613. 10.1046/j.1525-1497.2001.016009606.x 11556941 PMC1495268

[70] Kroenke K, Spitzer RL, Williams JBW, Löwe B. The Patient Health Questionnaire Somatic, Anxiety, and Depressive Symptom Scales: A systematic review. Gen Hosp Psychiatry. 2010;32: 345–359. 10.1016/j.genhosppsych.2010.03.006 20633738

[71] Henkel V, Mergl R, Kohnen R, Allgaier A-K, Möller H-J, Hegerl U. Use of brief depression screening tools in primary care: consideration of heterogeneity in performance in different patient groups. Gen Hosp Psychiatry. 2004;26: 190–198. 10.1016/j.genhosppsych.2004.02.003 15121347

[72] Williams JW, Pignone M, Ramirez G, Stellato CP. Identifying depression in primary care: a literature synthesis of case-finding instruments. Gen Hosp Psychiatry. 2002;24: 225–237. 10.1016/s0163-8343(02)00195-0 12100833

[73] Löwe B, Kroenke K, Herzog W, Gräfe K. Measuring depression outcome with a brief self-report instrument: sensitivity to change of the Patient Health Questionnaire (PHQ-9). J Affect Disord. 2004;81: 61–66. 10.1016/S0165-0327(03)00198-8 15183601

[74] Lucchesi F, Figueiredo MS, Mastandrea EB, Levenson JL, Smith WR, Jacinto AF, et al Physicians’ Perception of Sickle-cell Disease Pain. J Natl Med Assoc. 2016;108: 113–118. 10.1016/j.jnma.2016.04.004 27372471

[75] Ola BA, Yates SJ, Dyson SM. Living with sickle cell disease and depression in Lagos, Nigeria: A mixed methods study. Soc Sci Med. 2016;161: 27–36. 10.1016/j.socscimed.2016.05.029 27239705

[76] Tavakol M, Dennick R. Making sense of Cronbach’s alpha. Int J Med Educ. 2011;2: 53 10.5116/ijme.4dfb.8dfd 28029643 PMC4205511

[77] Streiner DL. Starting at the beginning: an introduction to coefficient alpha and internal consistency. J Pers Assess. 2003;80: 99–103. 10.1207/S15327752JPA8001_18 12584072

[78] Cronbach LJ. Coefficient alpha and the internal structure of tests. Psychometrika. 1951;16: 297–334.

[79] Halvorsrud L, Kirkevold M, Diseth A, Kalfoss M. Quality of Life Model: Predictors of Quality of Life Among Sick Older Adults. Res Theory Nurs Pract. 2010;24: 241–259. 10.1891/1541-6577.24.4.241 21197919

[80] Panepinto JA. Health-related quality of life in patients with hemoglobinopathies. Hematology Am Soc Hematol Educ Program. 2012;2012: 284–9. 10.1182/asheducation-2012.1.284 23233593

[81] McClish DK, Levenson JL, Penberthy LT, Roseff SD, Bovbjerg VE, Roberts JD, et al Gender differences in pain and healthcare utilization for adult sickle cell patients: The PiSCES Project. J women’s Heal. 2006;15: 146–154. 10.1089/jwh.2006.15.146 16536678

[82] Han S-H, Kim B, Lee S-A, Group KQ in ES. Contribution of the family environment to depression in Korean adults with epilepsy. Seizure. 2015;25: 26–31. 10.1016/j.seizure.2014.11.011 25645631

[83] Johnson EK, Jones JE, Seidenberg M, Hermann BP. The relative impact of anxiety, depression, and clinical seizure features on health‐related quality of life in epilepsy. Epilepsia. 2004;45: 544–550. 10.1111/j.0013-9580.2004.47003.x 15101836

[84] Cohen J. Statistical power analysis for the behavioral sciences 2nd edn Erlbaum Associates, Hillsdale; 1988.

[85] de Graaff B, Neil A, Sanderson K, Yee KC, Palmer AJ. Quality of life utility values for hereditary haemochromatosis in Australia. Health Qual Life Outcomes. 2016;14: 1–9. 10.1186/s12955-015-0404-4 26922941 PMC4770680

[86] Espallargues M, Czoski-Murray CJ, Bansback NJ, Carlton J, Lewis GM, Hughes LA, et al The impact of age-related macular degeneration on health status utility values. Invest Ophthalmol Vis Sci. 2005;46: 4016–4023. 10.1167/iovs.05-0072 16249475

[87] Peasgood T, Brennan A, Mansell P, Elliott J, Basarir H, Kruger J. The impact of diabetes-related complications on preference-based measures of health-related quality of life in adults with Type I diabetes. Med Decis Mak. 2016;36: 1020–1033. 10.1177/0272989X16658660 PMC5046160 27553209

[88] Pinto AM, Kuppermann M, Nakagawa S, Vittinghoff E, Wing RR, Kusek JW, et al Comparison and correlates of three preference-based health-related quality-of-life measures among overweight and obese women with urinary incontinence. Qual Life Res. 2011;20: 1655–1662. 10.1007/s11136-011-9896-5 21461953 PMC3174313

[89] Davison SN, Jhangri GS, Feeny DH. Comparing the Health Utilities Index Mark 3 (HUI3) with the Short Form‐36 Preference‐Based SF‐6D in Chronic Kidney Disease. Value Heal. 2009;12: 340–345. 10.1111/j.1524-4733.2008.00433.x 18657096

[90] Walters SJ, Brazier JE. What is the relationship between the minimally important difference and health state utility values? The case of the SF-6D. Health Qual Life Outcomes. 2003;1: 4 10.1186/1477-7525-1-4 12737635 PMC155547

[91] Khanna D, Furst DE, Wong WK, Tsevat J, Clements PJ, Park GS, et al Reliability, validity, and minimally important differences of the SF-6D in systemic sclerosis. Qual Life Res. 2007;16: 1083–1092. 10.1007/s11136-007-9207-3 17404896

[92] Rijken M, van Kerkhof M, Dekker J, Schellevis FG. Comorbidity of chronic diseases. Qual Life Res. 2005;14: 45–55. 10.1007/s11136-004-0616-2 15789940

[93] Berg B. Sf‐6d Population Norms. Health Econ. 2012;21: 1508–1512. 10.1002/hec.1823 22250070

[94] El-Shinnawy H, Goueli T, Nasreldin M, Meshref a. Anxiety, depressive disorders, and quality of life in adults with sickle cell disease. Middle East Curr Psychiatry. 2013;20: 80–86. 10.1097/01.XME.0000426319.48898.03

[95] Edwards R, Telfair J, Cecil H, Lenoci J. Self-efficacy as a predictor of adult adjustment to sickle cell disease: One-year outcomes. Psychosom Med. 2001;63: 850–858. 10.1097/00006842-200109000-00020 11573035

[96] Ellison AM, Shaw K. Management of vasoocclusive pain events in sickle cell disease. Pediatr Emerg Care. 2007;23: 832–841. 10.1097/PEC.0b013e31815a05e2 18007218

[97] Ballas SK. Update on pain management in sickle cell disease. Hemoglobin. 2011;35: 520–529. 10.3109/03630269.2011.610478 21910604

[98] Miller DR, Rogers WH, Kazis LE, Spiro A III, Ren XS, Haffer SC. patients’ Self‐report of Diseases in the Medicare Health Outcomes Survey Based on Comparisons With Linked Survey and Medical Data From the Veterans Health Administration. J Ambul Care Manage. 2008;31: 161–177. 10.1097/01.JAC.0000314707.88160.9c 18360178

[99] Feeny D, Spritzer K, Hays RD, Liu H, Ganiats TG, Kaplan RM, et al Agreement about identifying patients who change over time: cautionary results in cataract and heart failure patients. Med Decis Mak. 2012;32: 273–286. 10.1177/0272989X11418671 PMC3749910 22009666

[100] Richardson J, Iezzi A, Khan MA. Why do multi-attribute utility instruments produce different utilities: the relative importance of the descriptive systems, scale and ‘micro-utility’effects. Qual Life Res. 2015;24: 2045–2053. 10.1007/s11136-015-0926-6 25636660 PMC4493939

